# Meteorological and environmental factors associated with the exposure to tick-borne encephalitis virus (TBEV) in cattle, north-eastern France, 2018–2019

**DOI:** 10.1186/s13567-025-01588-8

**Published:** 2025-07-23

**Authors:** Laure Mathews-Martin, Raphaëlle Metras, Jean-Marc Boucher, Christophe Caillot, Sandrine A. Lacour, Marine Dumares, Cécile Beck, Gaëlle Gonzalez, Laure Bournez

**Affiliations:** 1 ANSES, Nancy Laboratory for Rabies and Widlife, 54220 Malzéville, France; 2 VetAgro Sup, ENSV-FVI, 69280 Marcy-L’Étoile, France; 3 ANSES, INRAE, ENVA, UMR Virology, ANSES Animal Health Laboratory, 94700 Maisons-Alfort, France; 4 ANSES, Laboratory for Food Safety, UVE, 94700 Maisons-Alfort, France; 5 INSERM, Institut Pierre Louis d’Epidémiologie Et de Santé Publique (IPLESP), Sorbonne Université, 75012 Paris, France

**Keywords:** Tick-borne encephalitis virus, France, cattle seroprevalence, spatial distribution, risk factors, random forest, modelling

## Abstract

Tick-borne encephalitis virus (TBEV) is a severe neurological disease that can be transmitted to humans through the bites of infected ticks or the consumption of unpasteurised dairy products from infected but asymptomatic ruminants. The recent detection of food-borne cases in France is a rising concern, since the production and consumption of raw milk cheese is common. There is limited data available on seroprevalence and factors associated with the exposure to TBEV of domestic ungulates in Europe, and to date, such data are not available in France. A total of 4,483 cattle sera were collected between 2018 and 2019. We used principal component analysis and spatial random forest modelling to explore meteorological and landscape predictors and their relationships with seroprevalence levels. TBEV antibodies were detected in cattle across the region, with an overall apparent seroprevalence of 7.5% (95% CI 6.7–8.3%). The highest seroprevalence was observed in the southern Vosges Mountains, reaching 72.5%. Cattle exposure was higher in areas where the annual land surface temperature was below 12 °C, mixed forest coverage exceeded 25%, and pastures located within 50 m of wooded areas covered more than 3%. This study represents the first large-scale serological survey of TBEV in cattle in France, revealing that TBEV is widespread in north-eastern France and extends beyond the distribution of TBE human cases. The main factors identified as influencing cattle exposure can be used to predict the risk of TBEV food-borne transmission. Further research is needed to fully understand this risk in France, including investigations into breeding and cheese practices.

**Supplementary Information:**

The online version contains supplementary material available at 10.1186/s13567-025-01588-8.

## Introduction

Tick-borne encephalitis (TBE) is the most prevalent arbovirosis in Europe with over 3000 cases reported per year [1]. TBE is caused by TBE virus (TBEV), which belongs to the *Flaviviridae* family and the genus *Orthoflavivirus* [2]. The European subtype (TBEV-Eu) is responsible for severe neuroinfections in humans, with a fatal outcome in 0.5 to 2% of cases. The majority of human infections result from tick bites, although approximately 1% of cases are attributed to the consumption of contaminated raw milk products [3]. Infected ruminants are usually asymptomatic but can shed the virus in their milk for up to 23 days [4]. Most foodborne cases are associated with raw dairy products from goats, followed by sheep and then cattle [3].

The virus naturally circulates between its vector, *Ixodes ricinus*, and rodents such as *Apodemus flavicollis* and *Clethrionomys glareolus* [2]. The virus is transmitted to ticks either systematically during the brief viremic period in rodents (2–9 days), or non-systemically by co-feeding, where at least one infected nymph transmits the virus to one or more uninfected larvae feeding nearby on the same rodent [5, 6]. The virus is then transmitted transtadially to the next life stage, with limited transovarian transmission [2]. The distribution of TBEV is more restricted than that of its vector, particularly in western Europe. Ecological factors that influence its distribution are not fully understood, despite a recent increase in studies on this topic [7–11]. Indeed, TBEV circulation and persistence require specific biotic and abiotic factors that differ from those needed for its vector or other tick-borne pathogens.

In France, the first cases of TBE were identified in 1968 in Alsace-Lorraine (north-eastern France). National incidence remains low, with a yearly notification rate of autochthonous TBE cases of 0.02 per 100,000 inhabitants from 2012 to 2020, although there has been a trend towards an increase since 2016 [1, 12]. The distribution of the virus is also poorly known in France, even in the historical endemic region of Alsace-Lorraine. Risk areas have been identified in Alsace, where human cases are regularly detected. A few cases have been identified in Lorraine, all near the Alsace border [13]. Nevertheless, the presence of TBEV is suspected further west (in the western part of the Grand-Est region) based on serological survey results from forestry workers [14]. In June 2020, the first foodborne TBE cases were reported in the Auvergne-Rhones-Alpes region, located in central-eastern France [15], where tick-borne cases had been reported since 2002–2003 [2, 12, 13, 16]. From May 2021 to May 2023, 6.5% of the 62 autochthonous French cases reported were suspected to have been transmitted through food [13]. These observations raise concerns about the potential occurrence of TBE-foodborne outbreaks in France, where cheese production from raw milk represents a major source of income for dairy farmers. To date, no survey has been conducted to assess the prevalence of TBEV in dairy products.

Assessing the exposure of dairy ruminants to infected tick bites and identifying the associated factors are crucial first steps in evaluating the risk of TBE-foodborne transmission. To our knowledge, there is limited documentation on the factors associated with the seroprevalence of domestic ungulates, even in European countries with more documented TBE-foodborne cases. Suckler cattle are well distributed in France, graze outdoors and blood samples are collected annually as part of disease surveillance programs. Moreover, a previous study conducted in the Vosgian Mountains’ endemic region of France (Alsace) confirmed that suckler cattle could be highly exposed to TBEV [17]. Consequently, they could be used as sentinels to identify the extent of virus circulation and as proxies for the exposure of dairy ruminants to TBEV, although cattle-derived dairy products are not the main source of food-borne transmission.

In the present study, we conducted a serological survey in suckler cattle to (i) evaluate the virus distribution in the French historical endemic region of Alsace-Lorraine, (ii) estimate the level of cattle exposure to TBEV, and (iii) identify the most significant meteorological, vegetation and landscape factors associated with their exposure to the virus.

## Materials and methods

### Study area

**Figure 1:**
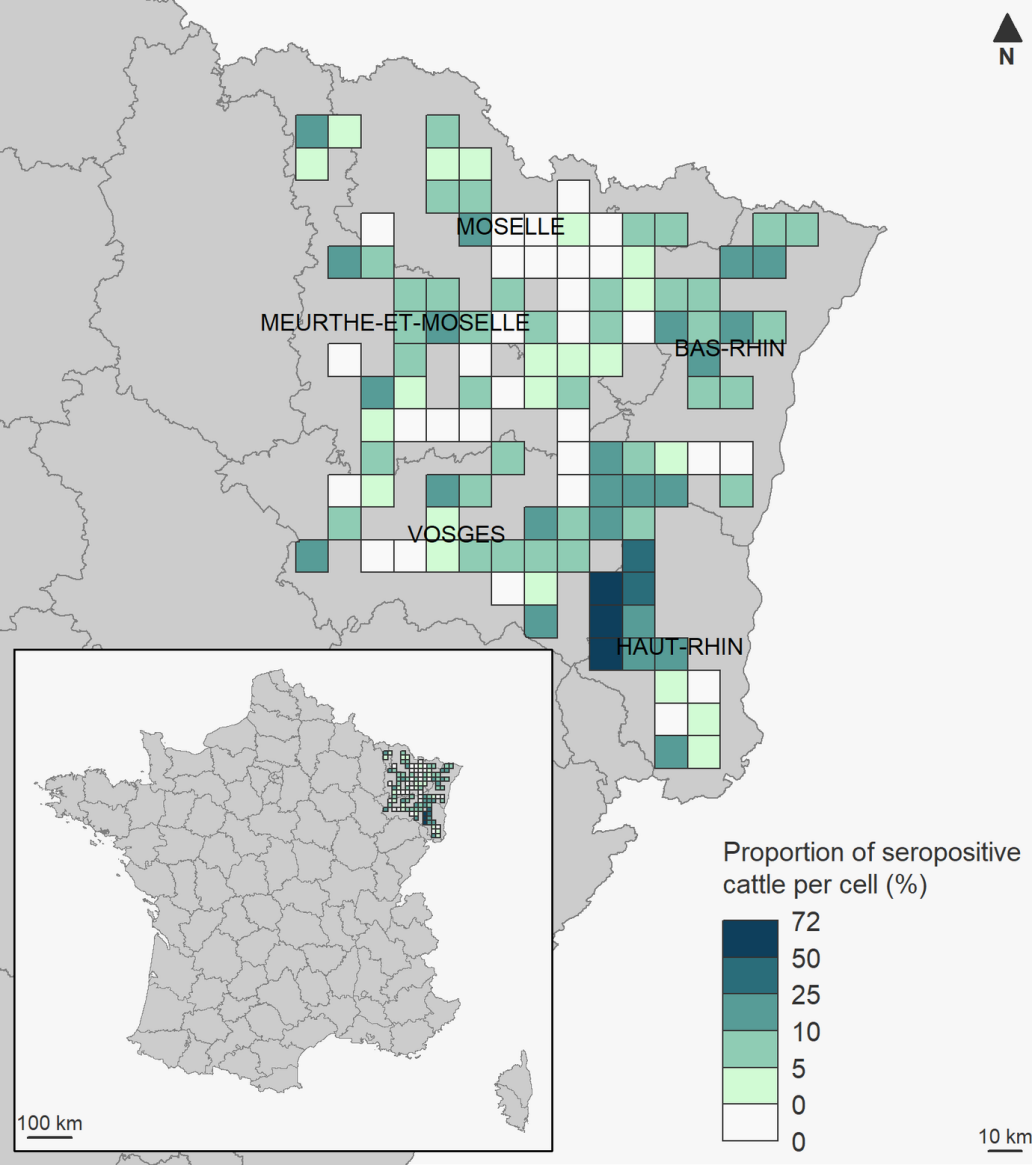
**Proportion of TBEV seropositive cattle per cell for the Grand-Est region, France, 2018–2019.** Meurthe-et-Moselle, Moselle and Vosges departments are in Lorraine region. Bas-Rhin and Haut-Rhin departments are in Alsace region.

The study was carried out in five departments (NUTS 3 administrative level) located in the northeastern region of France (Figure 1), within the Grand-Est region. From a grid of 10 × 10 km^2^ cells, 116 cells were randomly selected among those containing at least 100 suckler cattle and with at least 5% and 10% of the surface area covered by pasture and forest, respectively (Additionnal file 1). This selection ensured a minimum surface area suitable for *I. ricinus* ticks, TBEV and their hosts. The spatial resolution of the grid was defined to account for the average distance between pastures within individual farms. Since the median of the mean distances per farm between the centroid of each pasture and the centroid of the convex hull encompassing all pastures associated with that farm was 1.4 km, with 95% of values falling below 7.5 km (“Relevé Parcellaire Graphique” of 2018), we considered a 10 × 10 km grid resolution to be appropriate for representing cattle movements between pastures.

### Sampling strategy and serological testing

The sera were obtained from the serum libraries of departmental veterinary laboratories, as part of the national surveillance campaign on brucellosis and infectious bovine rhinotracheitis (IBR) conducted between 2017 and 2019, which targeted suckler cattle older than 24 months. An initial study tested 9819 sera (80–100 by cells) for antibodies against orthoflaviviruses using the ID Screen^®^ West Nile Competition test (ID Vet, Montpellier, France) [18] (Additional file 1). Only sera of cattle which were in the same farm for at least the last three years before sampling, as recorded in the French cattle identification tracing system (*Base de Données Nationale d’Identification*, BDNI) were tested. A three-year period was chosen as a compromise to ensure a sufficient number of samples per cell (approximately 80) and based on available data regarding antibodies persistence in ruminants. However, the longevity of antibodies in naturally infected ruminants remains uncertain. One study reported that antibodies can persist for three to six years in goats and sheep repeatedly immunized with a human-adapted vaccine [19], whereas another study suggested that antibody levels remain detectable for less than one or two years in naturally infected animals [20]. Positive and doubtful results were then subjected to microvirus microneutralisation tests (MNTs) for TBEV (354 sera) and Usutu virus (139 sera, Additional file 1). Since only two samples were weakly positive for Usutu, we considered that cattle may have been only minimally exposed to Usutu in our study area.

As the sensitivity of cELISA ID Screen^®^ was later observed to be low [17], a random subsample of 40 sera per cell, totaling 4483 samples, was subsequently tested with Immunozym FSME IgG All Species (Progen, GmbH, Germany) (Additional file 2). The test was conducted in accordance with the manufacturer’s instructions. The positive threshold was adjusted to 68 Vienna units (VIEU/mL) to achieve a sensitivity of 63% and a specificity of 95%, based on the results of Mathews-Martin et al. [17]. Given that cattle were poorly exposed to Usutu virus, we considered that a positive result with Immunozym was likely due to TBEV.

For the analysis, we only used the second dataset of 4483 samples. The outcome variable was the seroprevalence per cell, calculated by dividing the number of positive samples by the total number of sera tested. To account for the sensitivity and specificity of the test, the true seroprevalence in the study area was estimated as follows:

$$TP = {{\left( {AP + Sp - 1} \right)} \mathord{\left/ {\vphantom {{\left( {AP + Sp - 1} \right)} {\left( {Se + Sp - 1} \right)}}} \right. \kern-0pt} {\left( {Se + Sp - 1} \right)}}$$

where TP, true prevalence, AP, apparent prevalence, Sp, specificity (95%), and Se, sensitivity (63%) [21].

### Explanatory variables

TBEV seroprevalence in cattle depends on both the density of infected ticks in the area and the frequency of contacts between ticks and cattle. We considered two main categories of covariates, meteorological and environmental related-variables, that influence both mechanisms. All covariate were calculated for each cell.

We selected temperature- and humidity-related variables since they impact tick survival, development, and questing activity, with ticks being sensitive to desiccation [22]. Furthermore, these conditions may directly affect the transmission of TBEV from nymphs to larvae by altering TBEV replication [23, 24], the period of activity between larvae, nymphs, and rodents, as well as the questing height of nymphs, thereby influencing the number of nymphs feeding alongside larvae on rodents [25, 26]. The mean annual and seasonal land surface temperature during the day (LST) and night period (LSTN) were calculated for each cell (Table 1, see details in Additional file 3). We also computed the absolute mean autumnal cooling and spring warming rates. Indeed, an abrupt autumn chill combined with a rapid spring warm-up has been hypothesised to favour the synchronisation of larval and nymphal activity [26], thereby facilitating the transmission of TBEV between ticks. As a proxy for desiccation stress on ticks, we calculated the maximum number of consecutive dry days annually. The mean available water capacity index was also chosen as another proxy for relative humidity [27].

**Table 1:** **Description of the 14 explanatory variables selected for statistical analysis.**

Explanatory variables	Per cell	Values median,mean [min–max]
AWC^a^	Available Water Capacity index: mean index of avalaible water in soil	0.11, 0.11 [0.09–0.13]
decid_F^b^	Proportion of area covered by deciduous forest (%)	22.0, 22.2 [1.0–53.3]
conifer_F^b^	Proportion of area covered by coniferous forest (%)	1.5, 9.7 [0.0–58.9]
mixed_F^b^	Proportion of area covered by mixed forest (%)	2.5, 7.6 [0.0–37.5]
mead_f^c,d^	Proportion of meadow surface area within 50 m ofwooded areas (%)	2.4, 2.8 [0.1–9.6]
FPD^d^	Forest Patches Density (No. of forest patches per ha)	0.12, 0.12 [0.01–0.26]
LST^e^	Average Land Surface day-time Temperature per year (°C)	15.9, 15.4 [10.8–18.3]
LSTN^e^	Average Land Surface Temperature during the night period per year (°C)	5.8–5.9 [4.8–7.6]
LSTN_aut^e^	Average autumal (october, November, December) Land Surface Temperature during night period (°C)	2.0, 2.1 [1.3–3.3]
cool_aut^e^	Average autumnal cooling rate: regression coefficient of average daytime temperature from August to October (absolute value)	0.21, 0.21 [0.13 -0.27]
warm_spr^e^	Average spring warming rate: regression coefficient of average daytime temperature from February to April	0.23–0.23 [0.19–0.28]
EVI^f^	Average Enhanced Vegetation Index per year	0.39–0.39 [0.29–0.44]
EVI_sum^f^	Average summer (July, August, September) Enhanced Vegetation Index	0.43–0.43 [0.30–0.51]
CDD^g^	Maximum average number of Consecutive Dry Days per year (No.)	25.5–25.2 [20.1–28.5]

^a^ESDAC—LUCAS 2015 accessed on 01/02/2022.

^b^BD FORET^®^ v2 (2013–2014) provided by the French National Institute of Geographic and Forest Information (IGN-F) accessed on 04/05/2022.

^c^Relevé Parcellaire graphique 2018 provided by the French National Institute of Geographic and Forest Information (IGN-F) accessed on 07/12/2001.

^d^Corine Land Cover 2018, accessed on 10/12/2021.

^e^MODIS from 01/01/2015 to 31/12/2018, temporal resolution 8 days, accessed on 23/05/2022.

^f^MODIS from 01/01/2015 to 31/12/2018, monthly temporal resolution, accessed on 23/05/2022.

^g^E-OBS / Copernicus from 01/01/2015 to 31/12/2018, annual temporal resolution, accessed on 23/05/2022.

We then selected vegetation- and landscape-related variables (Table 1) since habitat properties, such as vegetation cover and density, influence both tick abondance by buffering the macroclimate effects and tick host abundance by providing shelter and food [28]. Enhanced Vegetation Index (EVI) is an index of vegetation greeness and can also be used as a proxy for relative humidity near the ground surface [29]. We calculated the mean annual and seasonal EVI. Given that forested areas are particularly suitable for ticks and their hosts [30], we calculated the proportion of cell surface area covered by coniferous, mixed and deciduous forest. Forest margins represent an important ecotone for vector-borne diseases, particurlarly those for which rodents are competent hosts [31]. Furthermore, the connection between forest patches through ecological corridors has been shown to increase the dispersal of ticks by wild fauna [32]. Consequently, a fragmented forest area may increase the potential contacts between infected ticks and domestic ruminants. We calculated the Forest Patch Density (PDF) to assess the degree of forest fragmentation per cell. To account for the frequency of contact between ticks and cattle, we calculated the proportion of meadow surface area within 50 m of wooded areas. The details of the calculation for each variable are presented in Additional file 3.

Although the importance of wild fauna in the epidemiological cycle of TBEV is recognised, we did not incorporate such data into the model due to their unavaibility at the resolution of our study (see details in Additional file 3). We assume the presence of all three relevant species of forestry rodents (*Apodemus flavicollis*, *Apodemus sylvaticus*, *Clethrionomys glareolus*), as well as roe deer, throughout the study area.

Highly correlated seasonal and annual variables (Pearson correlation coefficient > of 0.80) were not included for the analysis (Additional file 4). Consequently, a set of 14 meteorological and environmental variables were included in the subsequent analysis (Table 1).

### Identification of the factors associated with the level of cattle exposure

First, a descriptive analysis was performed using Principal Component Analysis (PCA) with the package *factoextra*. The objective was to identify which variables contributed most to each principal component and were most effective in distinguishing cells with the highest seroprevalence from those with the lowest. For this purpose, cells were classified into four categories based on the observed seroprevalence: “L” class [0–5%[; “ML” class [5–15%[; “MH” class [15%–40[and “H” class (≥ 40%). The relationship between the seroprevalence categories and each axis was analysed by examining the centroids of individuals within the same seroprevalence category. The strength of the relationship was quantified by the R^2^ of the analysis of variance of the coordinates of each seroprevalence category, and the significance was tested using a F-test.

In a second step, accounting for the spatial structure of our data, we fit a spatial random forest (RF) model using the package *spatialRF* [33] to estimate how cattle TBEV seroprevalence per cell was influenced by the set of factors. RF has significant advantages. It avoids collinearity misinterpretation. Futhermore, it does not assume a specific form of the relationship between the response variable and the covariates and allows for modelling “threshold relationships” when they exist. We built a matrix of distance between the centroids of the cells. Spatial autocorrelation was computed from 10 000 to 50 000 m every 10 000 m. To improve model performance, we used the *rf_tuning* function to select appropriate values for three key Random Forest hyperparameters. The optimised hyperparameters were 500 trees, 20 variables to choose from on each split and a minimal node size of 5. The spatial RF model was repeated 100 times. We plotted the Moran’s Index of the residuals to assess whether spatial autocorrelation issues had been mitigated. We evaluated variable importance by using the permutation importance scores of the predictors with the *plot_importance* function. This metric is calculated by randomly permuting each variable (including spatial predictors generated by the model) across all trees, and measuring how much the prediction error (on the out-of-bag data) increases when the variable is permuted. We also plotted partial dependence curves for the main predictors by setting the other predictors to their median value. Model performance was evaluated through R squared and Root Mean Squared Error (RMSE) values when predicting the “out-of-bag” data (fraction of data not used to train individual trees), using the *print_performance* function.

All data extraction and processing were carried out using R^®^ software version 4.1.1^®^ (R Development Core Team 2021–08-10). The maps were built with the package *mapsf* [34].

## Results

### Seroprevalence per cell

The overall apparent seroprevalence using cELISA was 7.5% (95% CI 6.7–8.3%) with 335 out of 4,483 cattle tested positive. Seroprevalence varied per cell from 0.0% for 34 cells to 72.5% (median 5%, IQR 7.5%), including three cells where seroprevalence exceeded 50% (Figure 1). Cattle were exposed to TBEV throughout the entire study area, with higher exposure observed particularly in the eastern Vosges and western Haut-Rhin. When accounting for the sensitivity and specificity of the test, the true prevalence in the study area was estimated at 4.3% (95% CI 3.0–5.6%).

### Principal components analysis

**Figure 2:**
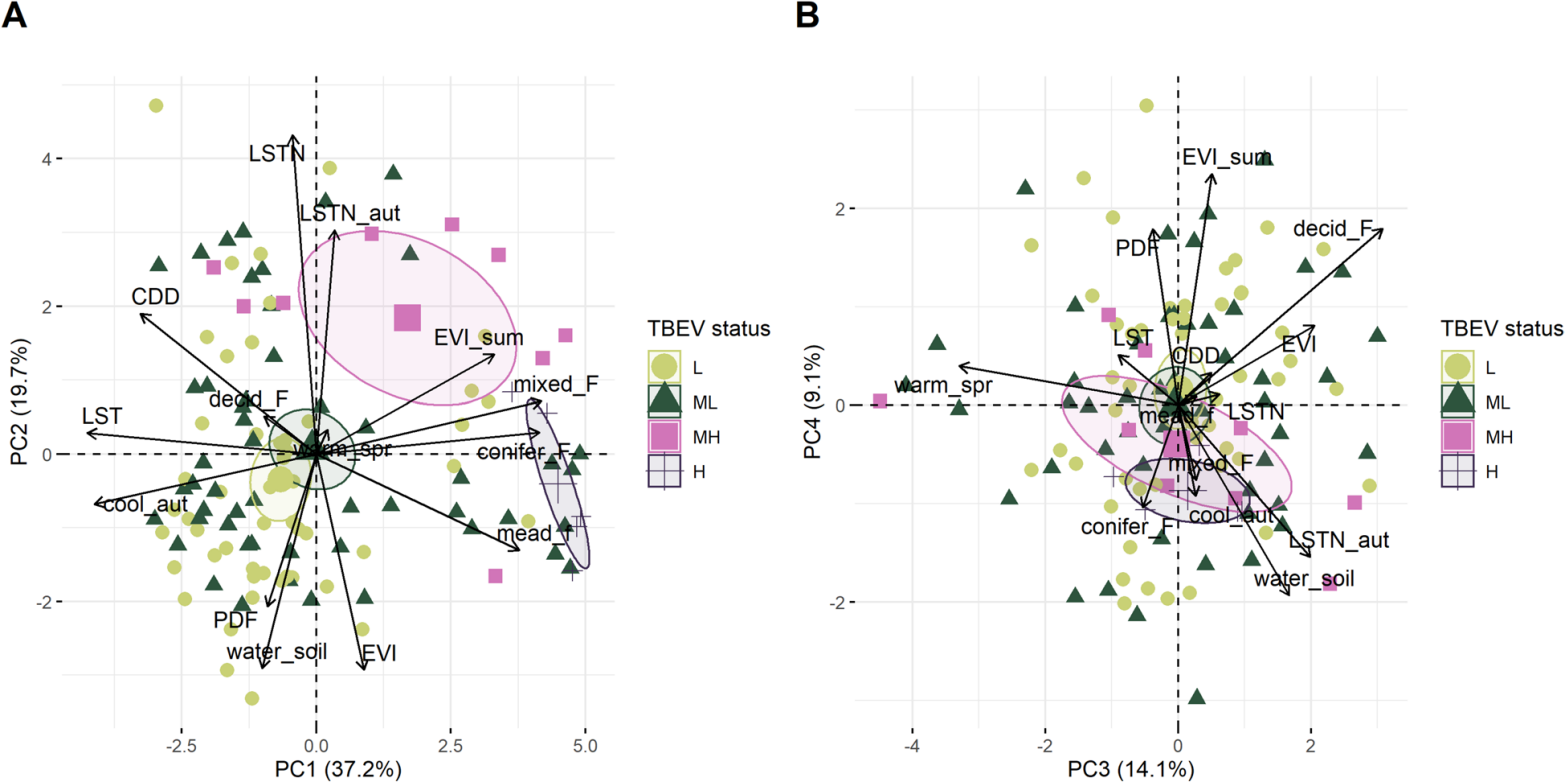
**Results of the principal component analysis on the contributions of variables to seroprevalence per cell**. Contribution of the meteorological, vegetation and landscape variables to (**A**) the principal components 1 (PC1) and 2 (PC2) and to (**B**) the principal components 3 (PC3) and 4 (PC4) and projection of cells according to the level of exposure of cattle with confidence ellipses (*L, Low seroprevalence [0–5%[*, *ML, Medium–Low: [5–15%[*, *MH, Medium High seroprevalence: [15–40%[, H, High seroprevalence: S* ≥ *40%)*. The horizontal axis corresponds to PC1 and PC3, and the vertical axis corresponds to PC2 and PC4. See Table 1 for the list of acronyms corresponding to the variables.

The first principal component (PC1) of PCA, explaining 37.2% of the variance, separated in some degree cells according to their seroprevalence (Figure 2; Additional files 5 and 6). Cells with the highest seroprevalence were mainly associated with a high proportion of the cell surface covered by mixed and coniferous forest, a lower mean annual day-time temperature, a mild decrease rate of temperatures during autumn and a higher proportion of meadow near wooded areas, compared to cells with the lowest seroprevalence (< 5%) (Figure 2A). To a lesser extent, the summer EVI also contributed to this first axis. Mean annual night-time temperature was the most important variable significantly correlated to the second principal component (PC2). This axis accounted for 19.7% of the variance, but did not contribute to distinguishing cells with the highest seroprevalence from those with the lowest. The main variable contributing to dimension 3, which accounted for 14.1% of the explained variance, was the proportion of deciduous forest (Figure 2B). Summer EVI contributed the most to the fourth axis (PC4, 9.1% of explained variance). However, neither the third axis nor the fourth one significantly discriminated exposure of cattle to TBEV per cell (Figure 2B).

### Random forest spatial analysis

**Figure 3:**
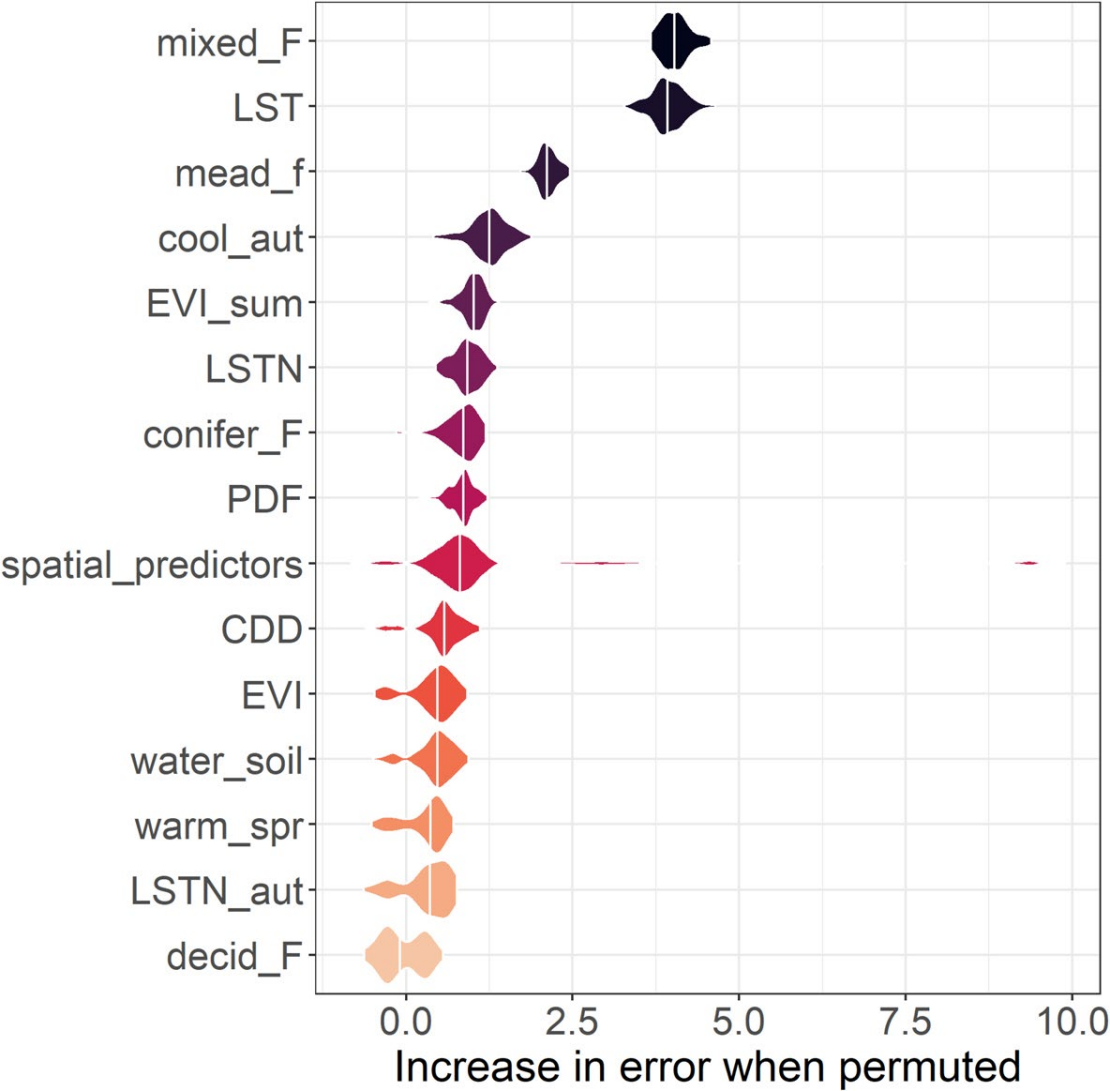
**Variable importance evaluated by the permutation performance score of each predictor using Random Forest model**. See Table 1 for the list of acronyms corresponding to the variables.

**Figure 4:**
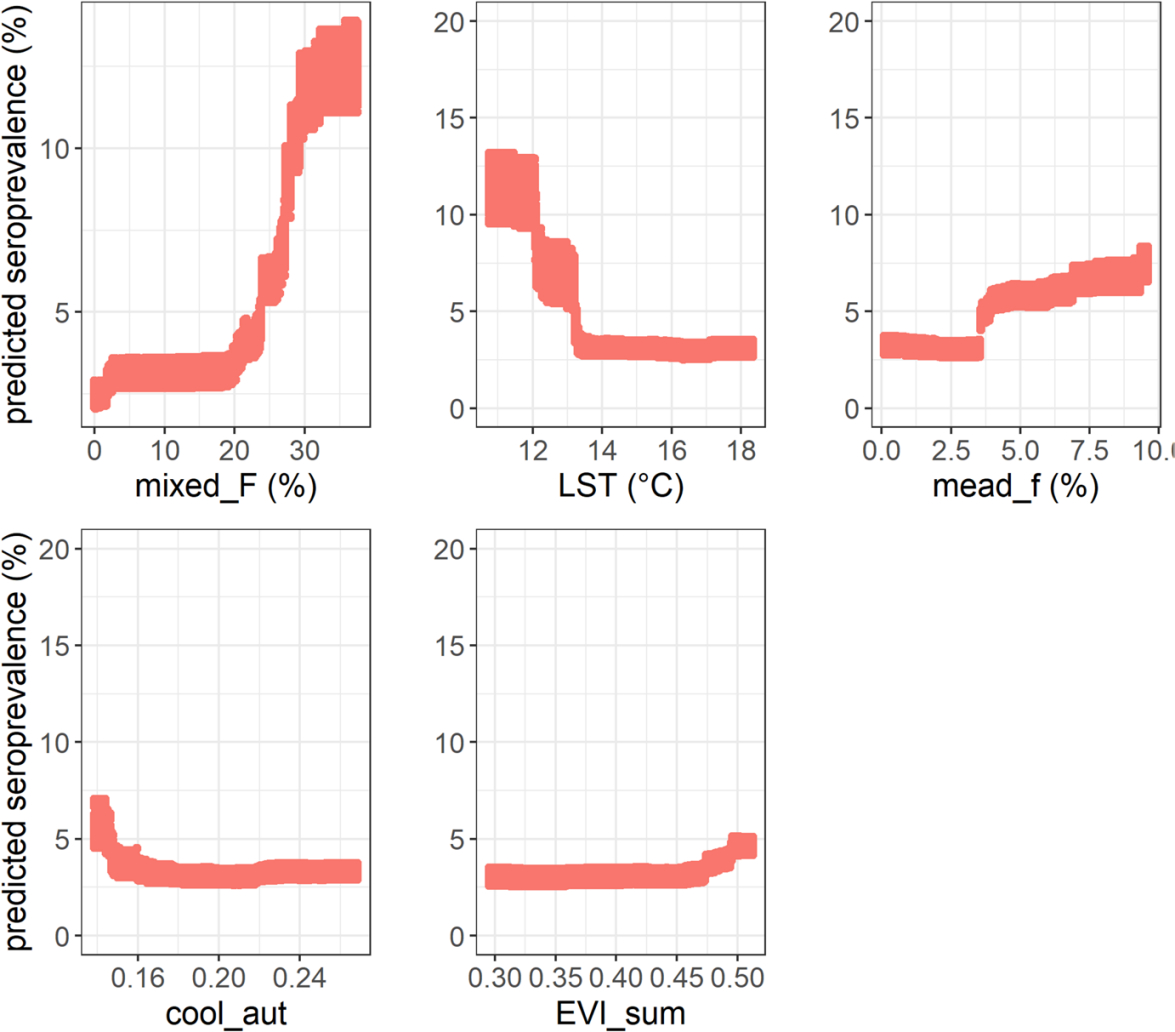
**Partial dependent curves for the five main predictors determined by the Random Forest model.** See Table 1 for the list of acronyms corresponding to the variables.

Based on the results of variable permutation scores, three main environmental and meteorological factors were associated with TBEV seroprevalence (Figure 3): mean annual day-time land surface temperature, proportion of mixed forest and proportion of meadow near wooded area. The partial dependence curves indicated a threshold rather than a linear relationship between the predictors and the proportion of seropositive cattle (Figure 4, Additional file 7). Cattle were more exposed in cells with a mixed forest cover above 28%, a mean annual day-time land surface temperature below 12.5 °C, and a proportion of meadow near forest above 3.5% (Figure 4). Furthermore, the models generated spatial predictors, some of which may prove crucial in explaining the observed variance (Figure 3). The model’s performance remained moderate, with the median proportion of variance explained by the predictors (R squared (oob)) being 0.64 (± 0.08) and the quantification of the errors (RMSE (oob)) being 6.76 (± 0.07). The residuals also showed significant slight autocorrelation up to 20 km (Additional file 8).

## Discussion

We conducted the first large-scale serological survey in cattle in France. The virus was present throughout the north-eastern study areas, contrary to the distribution of human cases. This corroborates findings from previous serological surveys of forestry workers exposed to TBEV, not only in Alsace but also in the departments of Lorraine [14]. This raises concerns about the under-diagnosis of human TBE cases in the Lorraine region. We therefore recommend more systematic testing of clinically suspected cases in patients from this area.

Although different serological tests were used, the overall cattle seroprevalence (true seroprevalence of 4.3%, 95% CI 3.0–5.6%) was lower than that reported in north-eastern Hungary (26.5%, 95% CI 21.5–32.2%; tested using EIA and seroneutralisation for positive and doubtful results), a country with a higher human incidence [35]. However, it was slightly higher than that observed in eastern Belgium (2.6%, 95% CI 1.4–3.8%; tested using seroneutralisation), a neighbouring country with low endemicity [36]. We did not confirm all positive results by seroneutralisation. We assumed that most seropositive cows had been exposed to TBEV, although we cannot exclude the possibility that some animals were exposed to other flavivirus, despite the low positivity rate found for Usutu virus. Cattle seroprevalence exhibited a spatial pattern in the study area, with over 50% of seropositive cattle in the southern part of the Vosgian Mountains. This is a mountainous region where cattle graze extensively in woodland areas and are likely highly exposed to tick bites. Despite our results suggesting that dairy grazing ruminants, such as suckler cattle, may be frequently exposed to TBEV in the study areas, no official food-borne TBE cases has been detected there. This is not surprising, given that the number of dairy goat and sheep farms—species whose raw milk products are most often implicated in TBEV food-borne contamination—is very limited in this region. Futhermore, while raw milk production from cows does exist, it is relatively uncommon. However, farmers who regularly consume raw milk from their own livestock may be at an increased risk of food-borne transmission. Conducting a dedicated risk assessment study would be valuable to evaluate the level of exposure among this population in the region.

We identified the principal factors associated with the level of cattle exposure to the virus in our study area. This is an essential first step in predicting domestic ruminant exposure and the risk of human foodborne-TBE cases. The results of the PCA and RF model were consistent and identified three most significant factors.

The percentage of surface area covered by mixed forest was the primary variable explaining model variance. Forests provide particularly suitable habitats for *I. ricinus* ticks and their hosts. Although mixed and/or coniferous forest areas were previously found positively associated with TBEV occurrence [37, 38], field studies have shown equivocal results concerning the abundance of questing ticks according to forest type [30, 39–41], reflecting the importance of other factors such as understorey vegetation. However, mixed forests are generally characterised by higher canopy closure due to greater complementarity in crown shape [42–44], and a constant presence of dense needle cover, which may maintain more favourable humidity conditions for tick development and survival, and for TBEV transmission cycle [23, 24]. More importantly, mixed forests may support higher abundances of small mammals than coniferous forests, particularly the yellow-necked mouse (*A. flavicollis*), which depends largely on tree seeds [45–47]. This species is considered a more competent host for TBEV transmission [48] and has been found associated to the presence of the virus [7]. Alternatively, the mixed forest variable may also be a confounding factor in relation to the study area, given that high-altitude areas are characterised by greater coverage of coniferous and mixed forest, as well as milder temperatures and higher humidity compared to the rest of the study area.

The proportion of meadow near forest was the third most important variable in our model. Wooded ecotones around pastures may act as favourable habitats for rodents and ticks, representing a significant source for tick presence in pastures [31]. Similarly, Rousseau et al. [49] observed that the proportion of forest in a buffer zone of one kilometre around pasture were associated with cattle seropositivity to *Anaplasma phagocytophilum*, a bacterial tick-borne disease. However, unlike this study and two others on TBE incidence [38, 50], forest fragmentation-related variable did not have significant effects in our results.

Low mean annual ground temperature during day-time was associated with high seroprevalence in cattle. Temperature-related variables are frequently found to be be associated with the presence of TBEV or the incidence of human cases in studies conducted at both continental and regional scales [7, 9, 11, 37, 38]. Consistent with our results, TBEV occurrence or human incidence has been observed to increase with lower mean temperatures (in winter across Europe [7]; in spring at a regional scale [37]) or with a higher number of frost days in winter (at a regional scale [9]). In our study, seasonal temperatures were strongly correlated with annual temperatures. Low temperatures in autumn, winter and early spring could potentially prolong the diapause behaviour of ticks, resulting in a later emergence of nymphs and a more synchronous activity between larvae and nymphs, thereby favouring TBEV transmission. Conversely, high temperatures in summer and autumn might decrease TBEV transmission by inducing a drop in virus titre in questing ticks [23, 24]. The relationship between TBEV circulation and temperature-related variables, as well as the specific mechanisms involved, requires further investigation to predict TBEV distribution.

To a lesser extent, a mild autumnal cooling rate was associated with higher cattle seroprevalence. This contrasts with previous studies, in which an abrupt autumnal chill was associated with TBEV presence [7, 10, 26, 51] and higher goat seroprevalence per pasture in Italy [52]. This unexpected observation may be related to the mountainous location of the study, where cattle might return to their barns earlier when temperatures decline rapidly in autumn, thus reducing their exposure to tick bites.

The performance of our model was only moderate, suggesting that some important variables were lacking, such as humidity or the abundance of forest rodents (*A. flavicollis* and *M. glareolus*) or cervids [7, 51]. In absence of these data at the scale of the study, EVI and available water capacity of soils were used as proxies for relative humidity, and landscape variables were used as proxies for host abundance. Furthermore, incorporating additional data on herd management practices that may influence cattle exposure levels, such as pasture location, grazing practices or acaricide treatments, might help to improve the models. Although no official data are available, acaricide treatments are likely to have minimal impacts as they are typically administrated sporadically. While a 10 × 10 km spatial resolution was selected to account for cattle movements between pastures, our estimates of cattle exposure may have been partially biased by the time animals spent outside the cell to which they were assigned. Some animals may have grazed in pastures located in adjacent cells and more rarely in pastures located farther away (which was the case for 2.3% of farms across the entire study area). Moreover, for animals that changed farms (though not within the three years preceding sampling), the time spent outside their cell may be relevant if the antibodies persist for longer than three years. In addition, within a given cell, the environmental characteristics surrounding the pastures used by the sampled animals may differ from the average conditions of all pastures in that cell—the variable used in our model—thereby potentially introducing bias into the results.

TBEV is widely distributed across the five departments in the north-eastern region of France, even in areas where no human TBE cases have yet been reported. Locally, cattle can be highly exposed to the virus. This geographic discrepancy raises concerns about under-diagnosis of human cases, particularly in regions such as Lorraine, and underscores the need for more systematic testing of patients with compatible symptoms. No foodborne-TBE human cases have been officially detected in the region, which may be related to the low importance of the local raw-milk industry. However, farmers who regularly consume raw milk from their own livestock could be at increased risk of foodborne transmission. A targeted risk assessment would be valuable to evaluate this potential exposure. Our study provides insights into the main factors influencing cattle exposure to TBEV, which can be useful for further research aiming to predict spatial risk of ruminants exposure to TBEV and of foodborne-TBE transmission.

## Supplementary Information

**Additional file 1. Pilot study.**

**Additional file 2. Number of cattle sera tested per cell.**

**Additional file 3.** **Meteorological, vegetation and landscape variables.**

**Additional file 4.** **Matrix of correlation of meteorological, vegetation and landscape variables.**

**Additional file 5.** **Percentage of variance explained by each dimension of the principal component analysis.**

**Additional file 6. Relationship between the qualitative variables “class of seroprevalence” and the axes and significance (F-test).**

**Additional file 7. Distribution of the main predictors in the study area.** **A** Proportion per cell (%) of surface area covered by mixed forest, **B** mean annual LST (°C) per cell, and **C** proportion per cell (%) of surface area of meadow neighboring forest in the 116 cells of the area.

**Additional file 8. Moran’s index of the residuals of the tuned and repeated Random Forest spatial model.** Spatial autocorrelation was not completely alleviated at a distance of 20 km.

## Data Availability

The dataset supporting the conclusions of this article is included within the article and its additional files.
