# Supplementary material for: Meteorological and environmental factors associated with the exposure to tick-borne encephalitis virus (TBEV) in cattle, north-eastern France, 2018–2019
Source: Vet Res. 2025 Jul 23;56:157. doi: 10.1186/s13567-025-01588-8 (PMC12288213; doi:10.1186/s13567-025-01588-8)
Supplement: Supplementary file 1 — Additional file 1. Pilot study. [file 13567_2025_1588_MOESM1_ESM.docx]

**Additional file 1. Pilot study**

Since the median of the mean distances per farm between the centroid of each pasture and the centroid of the convex hull encompassing all pastures associated with that farm was 1.4 km, with 95% of values falling below 7.5 km (“Relevé Parcellaire Graphique” of 2018), we considered a 10 × 10 km grid resolution to be appropriate for representing cattle movements between pastures. A grid of 207 10 × 10 km cells was created in the five departments (NUTS 3 administrative levels) of north-eastern France (Bas-Rhin, Haut-Rhin, Meurthe-et-Moselle, Moselle and Vosges). Among the 168 cells that contained at least 100 adult female suckling cattle according to the French cattle identification tracing system (*Base de Données Nationale d’Identification*, BDNI), at least 10% of the surface area covered by forest (BD Forêt v2® database (2013-2014, vector data),) and at least 5% of the surface covered by pasture (“Relevé Parcellaire Graphique” (RPG) of 2018), 116 cells were randomly selected. In each cell, between 80 and 100 serum sample of cattle per cell from different farms were also randomly selected from the serum libraries of veterinary laboratories, collected as part as a national brucellosis and infectious bovine rhinotracheitis (IBR) surveillance campaign in 2017-2019. Only sera from cattle that had remained on the same farm for at least the last three years before sampling were tested, as in the BDNI. A three-year period was chosen as a compromise to ensure a sufficient number of samples per cell (approximately 80) and based on available data regarding antibodies persistence in ruminants. However, the longevity of antibodies in naturally infected ruminants remains uncertain. One study reported that antibodies can persist for three to six years in goats and sheep repeatedly immunized with a human-adapted vaccine [19], whereas another study suggested that antibody levels remain detectable for less than one or two years in naturally infected animals [20].

Sera were tested with ID Screen® West Nile Competition (ID Vet, Montpellier France) to detect antibodies against orthoflaviviruses. Doubtful and positive results (adjusted threshold S/N ≤ 70%) were submitted to micro virus micro neutralization tests (MNTS, strain Hypr, GTenbank ID U39292.1) to confirm the presence of specific neutralising antibodies against TBEV. Sera with a result in S/N ≤ 50% (doubtful threshold recommended by the manufacturer) were also tested in MNT for USUTU virus (USUV) (strain Italy 2012, 206795-3/2012, Genbank ID KX816653.1, provided by Davide Lelli, IZSLER, Brescia). A sample was considered positive for TBEV and USUV, if cells were protected at a serum dilution of 1:20 and 1:10, respectively. Overall, 182/354 cELISA positive and doubtful sera (threshold S/N ≤ 70) tested in MNT for TBEV were positive, and only 2/139 were weakly positive for USUV (titre 1:20), but negative for TBEV. Therefore, we considered that cattle may have only been weakly exposed to USUV in our study area.
