# Supplementary figures and images for: Meteorological and environmental factors associated with the exposure to tick-borne encephalitis virus (TBEV) in cattle, north-eastern France, 2018–2019
Source: Vet Res. 2025 Jul 23;56:157. doi: 10.1186/s13567-025-01588-8 (PMC12288213; doi:10.1186/s13567-025-01588-8)

**Additional file 2. Number of cattle sera tested per cell**


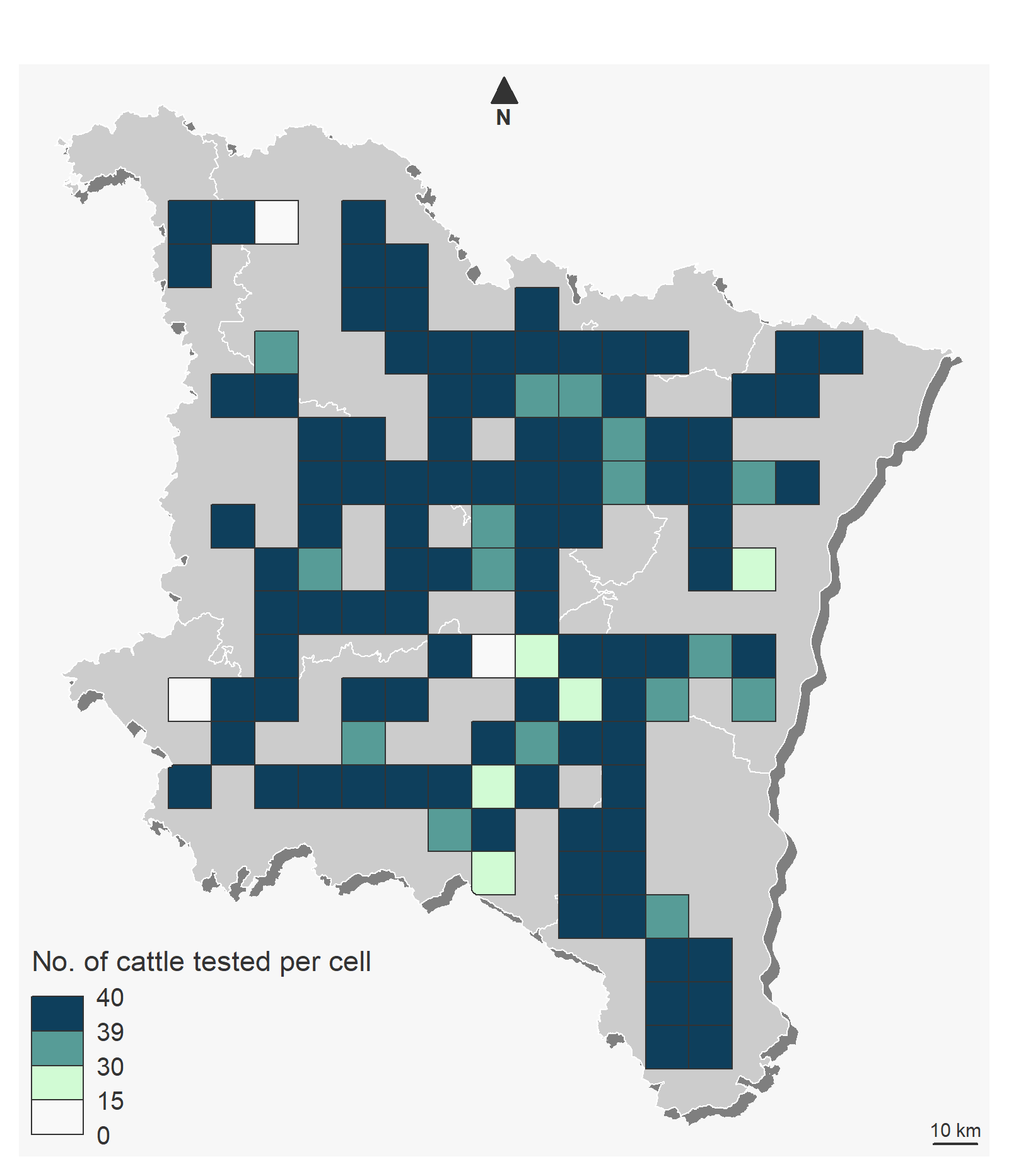

Supplement: Supplementary file 2 — Additional file 2. Number of cattle sera tested per cell. [file 13567_2025_1588_MOESM2_ESM.docx]
