# Supplementary material for: Meteorological and environmental factors associated with the exposure to tick-borne encephalitis virus (TBEV) in cattle, north-eastern France, 2018–2019
Source: Vet Res. 2025 Jul 23;56:157. doi: 10.1186/s13567-025-01588-8 (PMC12288213; doi:10.1186/s13567-025-01588-8)
Supplement: Supplementary file 3 — Additional file 3. Meteorological, vegetation and landscape variables. [file 13567_2025_1588_MOESM3_ESM.docx]

**Additional file 3. Meteorological, vegetation and landscape variables**

In order to ascertain the meteorological and vegetation variables, we have chosen to process them from 2015 to 2018. This is on the assumption that the level of seroprevalence is influenced by conditions observed in the three to four years prior to blood sampling. Temperature variables were processed from Land Surface Temperature/Emissivity 8-Day L3 Global 1km ([Wan, Z., Hook, S., Hulley, G. (2015). MOD11A2 MODIS/Terra Land Surface Temperature/Emissivity 8-Day L3 Global 1km SIN Grid V006. NASA EOSDIS Land Processes DAAC. Accessed 2022-06-07 from https://doi.org/10.5067/MODIS/MOD11A2.006. Accessed June 7, 2022.](https://doi.org/10.5067/MODIS/MOD11A2.006)) for both Land Surface Temperature during daytime (LST) and during night period (LSTN). They were downloaded from Moderate Resolution Imaging Spectroradiometer database (MODIS - MOD13A3 Version 6), with a spatial resolution of 1 km and a temporal resolution of eight days from January the 1^st^ 2015 to December 31^th^ 2018 over an area covering the five departments of interest in the Grand-Est region. Furthermore, we calculated the average autumnal cooling rate as the regression coefficient of average daytime temperature (LST) from August to October. We proceeded with the absolute values for statistical analysis. Similarly, the spring warming rate was calculated as the regression coefficient of average daytime temperature form February to April.

Considering index of vegetation, we chose EVI (Enhanced Vegetation Index takes into account the leaf surface, the quantity of chlorophyll and the structure of the vegetation cover) rather than NDVI (Normalized Difference Vegetation Index) data. The latter is more sensitive to atmospheric conditions, sun angle and soil type under the vegetation (Matsushita B. et al. 2007). EVI minimizes variations between the canopy and the ground and improves sensitivity under dense vegetation conditions, although it remains more sensitive to topography. However, this effect diminishes or even disappears when the pixel size resolution increases, and we chose a resolution of 1 km instead of 250 m. The Vegetation Indices Monthly L3 Global 1 km product (product ([Didan, K. (2015). MOD13A3 MODIS/Terra vegetation Indices Monthly L3 Global 1km SIN Grid V006. NASA EOSDIS Land Processes DAAC. Accessed 2022-05-23 from https://doi.org/10.5067/MODIS/MOD13A3.006. Accessed May 23, 2022.](https://doi.org/10.5067/MODIS/MOD13A3.006))) were downloaded from MODIS database (MODIS - MOD13A3 Version 6), with a spatial resolution of 1 km and a temporal resolution of one month from January the 1^st^ 2015 to December 31^th^ 2018 over the same area as for LST and LSTN data.

The maximum annual number of consecutive dry days (CDD) was downloaded at the spatial resolution of 0.1° (~9 km²) E-OBSv23 from the European database Copernicus and averaged between 2015 and 2018. For one cell, we replaced the missing value of this variable with the mean value of the variable in the three nearest cells.

Available water capacity index (AWC) derives from the texture of the soil in relation with the combination of the amounts of silt, sand and clay. Data was extracted from the topsoil database provided by the European Soil Data Centre (ESDAC) and stored in a raster file of 500 × 500 m pixels describing soil composition (20 cm topsoil). This database was built upon the Land Use and Cover Area frame Statistical survey (LUCAS) (Ballabio et al. [27]).

Forest data were extracted from the BD Forêt v2® database (2013-2014, vector data), provided by the French National Institute of Geographic and Forest Information (IGN). It describes the forest and natural plant formations by a land cover approach from areas larger than 5000 m². Data from BD Forêt v2® are elaborated by visual interpretation of infrared images and were updated in 2013 for the department of Vosges and in 2014 for the four others. We retrieved data for deciduous forest (TFV_G11: “closed deciduous forest” and “open deciduous forest”), mixed forest (TFV_G11: “closed mixed forest” and “open mixed forest”) and coniferous forest (TFV_G11: “closed coniferous forest” and “open coniferous forest”).

To calculate the proportion of meadow within 50 m from wooded areas, grassland locations were extracted from the “Relevé Parcellaire Graphique” (RPG) of 2018 (vector data of agricultural parcel contours) provided by the IGN. We included: summer pastures and moors (code_group 17), natural grasslands and temporary grasslands older than 5 years (code_group 18) and temporary grasslands (code_group 19). We also used forest data from the classified remote sensing data Corine Land Cover database (raster data of Copernicus, 2018) with a spatial resolution of 5 000 m². Forests included broadleaved, coniferous and mixed (CLC 2018 codes 311, 312 and 313).

The *landscapemetrics* package was used to calculate the forest patch density per cell

The set of dependent variables did not include biotic data related to host populations. Our objective was to calculate a proxy for the density of red deer and deer per km² of forest per cell using data from hunted animals provided by the departmental hunting federations and/or the departmental territory directions. Unfortunately, the hunting federations in Bas-Rhin and Haut-Rhin do not have registers for roe deer. To fill in the missing information, we compared the results obtained for the three other departments with the presence index modelled by Alexander et al. (Alexander et al. 2014). As there was poor correlation between the two sets of data for the 93 cells where both data were available, we did not include this index in our model. Additionally, we did not include biotic data related to forest rodents (*Apodemus sylvaticus*, *Myodes glareolus* and *Apodemus flavicollis*). The INPN (National Inventory of Natural Heritage) reports the presence of these three species in all five departments of our study, but without more detailed information at a smaller scale (https://inpn.mnhn.fr/espece/cd_nom/61510/tab/carteaccessed 24 may 2023).
