# Supplementary material for: Meteorological and environmental factors associated with the exposure to tick-borne encephalitis virus (TBEV) in cattle, north-eastern France, 2018–2019
Source: Vet Res. 2025 Jul 23;56:157. doi: 10.1186/s13567-025-01588-8 (PMC12288213; doi:10.1186/s13567-025-01588-8)

**Additional file 4. Matrix of correlation of meteorological, vegetation and landscape variables**


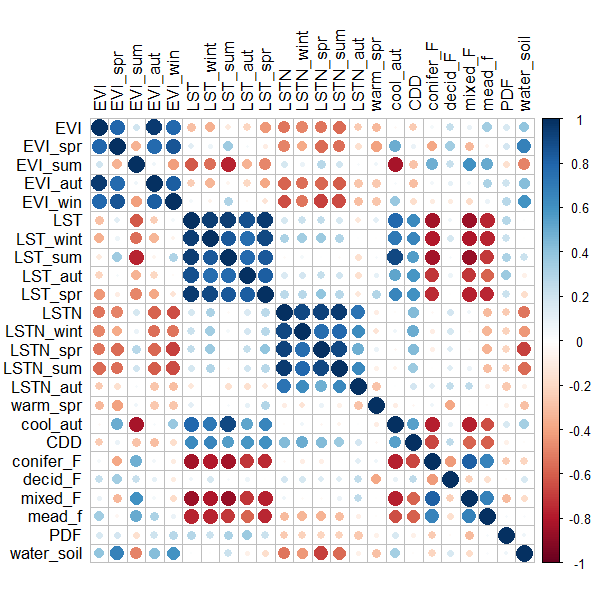

Supplement: Supplementary file 4 — Additional file 4. Matrix of correlation of meteorological, vegetation and landscape variables. [file 13567_2025_1588_MOESM4_ESM.docx]
