# Supplementary material for: Meteorological and environmental factors associated with the exposure to tick-borne encephalitis virus (TBEV) in cattle, north-eastern France, 2018–2019
Source: Vet Res. 2025 Jul 23;56:157. doi: 10.1186/s13567-025-01588-8 (PMC12288213; doi:10.1186/s13567-025-01588-8)
Supplement: Supplementary file 6 — Additional file 6. Relationship between the qualitative variables “class of seroprevalence” and the axes and significance (F-test). [file 13567_2025_1588_MOESM6_ESM.docx]

**Additional file 6. Relationship between the qualitative variables “class of seroprevalence” and the axes and significance (F-test)**

| **Class of seroprevalence** | **Axis 1** | | **Axis 2** | | **Axis 3** | |
| --- | --- | --- | --- | --- | --- | --- |
|  | **estimate** | ***p*-value** | **estimate** | ***p*-value** | **estimate** | ***p*-value** |
| L | 2.0 | *** | -0.6 | * | NC | NC |
| ML | NC | NC | NC | NC | NC | NC |
| MH | -0.3 | *** | -1.6 | *** | NC | NC |
| H | -3.1 | *** | NC | NC | NC | NC |

H, High seroprevalence: S ≥ 40%, L, Low seroprevalence [0%-5%[, MH, Medium High seroprevalence: [15%-40, ML, Medium-Low: [5%-15%[, %[,; NC: not correlated; **p*-value<0.05, ****p*-value <<<0.05;
