# Supplementary material for: Meteorological and environmental factors associated with the exposure to tick-borne encephalitis virus (TBEV) in cattle, north-eastern France, 2018–2019
Source: Vet Res. 2025 Jul 23;56:157. doi: 10.1186/s13567-025-01588-8 (PMC12288213; doi:10.1186/s13567-025-01588-8)
Supplement: Supplementary file 7 — Additional file 7. Distribution of the main predictors in the study area. A Proportion per cell (%) of surface area covered by mixed forest, B mean annual LST (°C) per cell, and C proportion per cell (%) of surface area of meadow neighboring forest in the 116 cells of the area. [file 13567_2025_1588_MOESM7_ESM.docx]

**Additional file 7. Distribution of the main predictors in the study area.** (A) Proportion per cell (%) of surface area covered by mixed forest, (B) mean annual LST (°C) per cell, and (C) proportion per cell (%) of surface area of meadow neighboring forest in the 116 cells of the area.

**
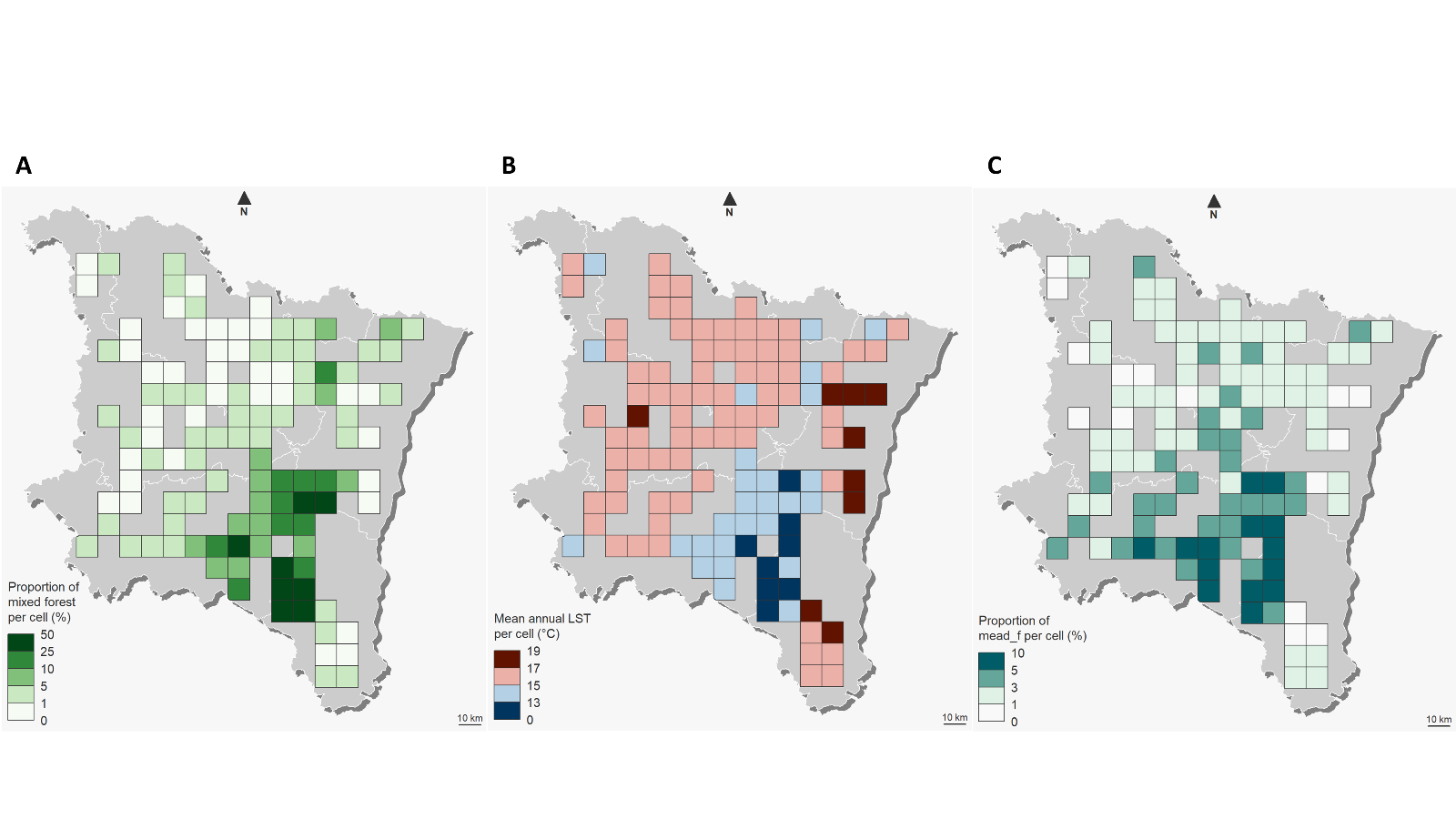
**
