# Supplementary material for: Meteorological and environmental factors associated with the exposure to tick-borne encephalitis virus (TBEV) in cattle, north-eastern France, 2018–2019
Source: Vet Res. 2025 Jul 23;56:157. doi: 10.1186/s13567-025-01588-8 (PMC12288213; doi:10.1186/s13567-025-01588-8)

**Additional file 8. Moran’s index of the residuals of the tuned and repeated Random Forest spatial model.** Spatial autocorrelation was not completely alleviated at a distance of 20 km.


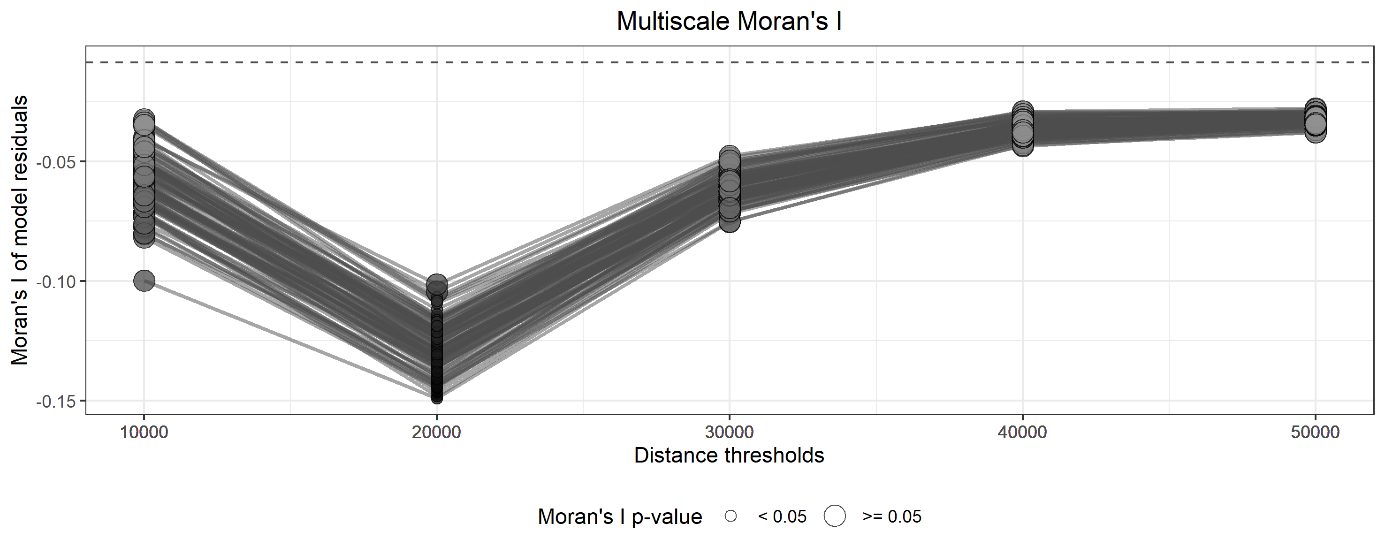

Supplement: Supplementary file 8 — Additional file 8. Moran’s index of the residuals of the tuned and repeated Random Forest spatial model. Spatial autocorrelation was not completely alleviated at a distance of 20 km. [file 13567_2025_1588_MOESM8_ESM.docx]
